# Case report: Severe central nervous system manifestations associated with aberrant efavirenz metabolism in children: the role of *CYP2B6* genetic variation

**DOI:** 10.1186/s12879-016-1381-x

**Published:** 2016-02-02

**Authors:** Francoise Pinillos, Collet Dandara, Marelize Swart, Renate Strehlau, Louise Kuhn, Faeezah Patel, Ashraf Coovadia, Elaine Abrams

**Affiliations:** 1 Empilweni Services and Research Unit (ESRU), Rahima Moosa Mother and Child Hospital, Department of Paediatrics and Child Health, Faculty of Health Sciences, University of the Witwatersrand, Johannesburg, South Africa; 2 Pharmacogenetics and Cancer Research Group, Division of Human Genetics, Department of Pathology & Institute of Infectious Disease and Molecular Medicine, University of Cape Town, Cape Town, South Africa; 3 Gertrude H. Sergievsky Center, College of Physicians and Surgeons; and Department of Epidemiology, Mailman School of Public Health, Columbia University, New York, NY USA; 4 ICAP, Mailman School of Public Health, and College of Physicians & Surgeons Columbia University, 722 W168th street, New York, NY 10032 USA

**Keywords:** Efavirenz, Paediatrics, CYP2B6, HIV, Paediatric HIV, Antiretroviral treatment, CNS, Seizures, Cerebellum

## Abstract

**Background:**

Efavirenz, widely used as part of antiretroviral drug regimens in the treatment of paediatric human immunodeficiency virus infection, has central nervous system side effects. We describe four children presenting with serious, persistent central nervous system adverse events who were found to have elevated plasma efavirenz concentrations as a result of carrying *CYP2B6* single nucleotide polymorphisms, known to play a role in the metabolism of EFV. None of the children had a *CYP2B6* wildtype haplotype. We believe this is the first case of cerebellar dysfunction associated with efavirenz use to be described in children.

**Case presentation:**

Four black African children, between the ages of 4 and 8 years presenting between 1 and 20 months post-efavirenz initiation, are described. Cerebellar dysfunction, generalised seizures and absence seizures were the range of presenting abnormalities. Plasma efavirenz levels ranged from 20-60 mg/L, 5–15 times the upper limit of the suggested reference range. All abnormal central nervous system manifestations abated after efavirenz discontinuation.

**Conclusion:**

Efavirenz toxicity should always be considered in human immunodeficiency virus-infected children with unexplained central nervous system abnormalities. Our findings further our understanding of the impact of genetic variants on antiretroviral pharmacokinetics in children across various ethnic groups. Screening for potential EFV-toxicity based on the *CYP2B6 c.516* SNP alone, may not be adequate.

## Background

Efavirenz (EFV) is a potent non-nucleoside reverse transcriptase inhibitor (NNRTI) used as part of combination antiretroviral (ARV) regimens in the treatment of paediatric and adult human immunodeficiency virus (HIV) infection. Central nervous system (CNS) complications associated with EFV use have been well characterised in adults, but are less commonly described in children. We report on four children who presented with serious, persistent CNS adverse effects attributed to EFV toxicity while receiving standard recommended weight-based EFV dosing as per South African guidelines. All four children were found to have plasma EFV concentrations above the suggested therapeutic range and carried *CYP2B6* single nucleotide polymorphisms (SNPs), *CYP2B6 c.516G > T*, *CYP2B6 c.785A > G* and *CYP2B6 c.983 T > C*, which are associated with reduced *CYP2B6* function and decreased EFV metabolism Table 1.

**Table 1:** Case detail summary

	[Case 1]^b^	[Case 2]^b^	[Case 3]^a^ ^b^	[Case 4]^b^
CNS Adverse Event	Generalised tonic-clonic seizures Aggressive behavior	Acute progressive ataxia, with cerebellar signs Anti-social behavior	Generalised tonic-clonic seizures Progressively poor school performance	Absence seizures
Event Described	Generalised tonic-clonic seizure	Cerebellar dysfunction	Generalised tonic-clonic seizure	Absence seizure
Sex	Male	Female	Female	Female
Starting ART Regimen	LPV/r, 3TC, d4T	LPV/r, 3TC, d4T	LPV/r, 3TC, d4T	RTV, 3TC, d4T
Age at ART start (months)	4.9	2.4	16.7	4
ART regimen at first event	EFV, 3TC, ABC	EFV, 3TC, ABC	EFV, 3TC, ABC	EFV, 3TC, d4T
Time on EFV at first event (months)	3.3	19.8	13.7	0.9
Age at first event	4 years 6 months	4 years 10 months	7 years 6 months	4 years 7 months
EFV dose at first event (mg/kg/dose)	21 mg/kg/dose	21 mg/kg/dose	15 mg/kg/dose	21 mg/kg/dose
Age at second event	5 years	4 years 11 months	9 years 5 months	N/A
Time on EFV at second event (months)	9.6	21.3	37.9	N/A
Time on EFV at time of drug level (months)	10.1	23.5	49.7	1.2
EFV level mg/L (reference range)	20 mg/L (1-4 mg/L)	60.54 mg/L (Ref > 1 mg/L)	51.23 mg/L (1-4 mg/L)	19.62 mg/L (1-4 mg/L)
Time since last dose prior to levels (hours)	13 h post dose	13 h post dose	14 h post dose	15 h post dose
Genotype	Heterozygous *CYP2B6 516G/T* Heterozygous *CYP2B6 785A/G*	Heterozygous *CYP2B6 516G/T* Heterozygous *CYP2B6 983 T/C*	Heterozygous *CYP2B6 516G/T* Heterozygous *CYP2B6 983 T/C*	Homozygous *CYP2B6 516 T/T*

^a^Neverest 2 clinical trial (ClinicalTrials.gov, NCT00117728) [3] was a NVP-conserving strategy, aiming to preserve regimens for children exposed to NVP as part of Prevention of Mother-to-Child Transmission (PMTCT). Children were either randomized to continue on a protease inhibitor (PI) or switch to NVP

^b^Neverest 3 clinical trial (ClinicalTrials.gov, NCT01146873) [1] evaluated PI-sparing treatment strategies among NVP-exposed HIV infected children initially treated with lopinavir/ritonavir (LPV/r), but were either randomized to stay on LPV/r or switch to EFV

Abbreviations: *LPV/r* (lopinavir/ritonavir), *3TC* (lamivudine), *d4T* (stavudine), *EFV* (efavirenz), *RTV* (ritonavir), *ABC* (abacavir), *CYP2B6* (Cytochrome P450 2B6), *G* (guanine), *T* (thymine), *A* (adenine), *C* (cytosine)

## Case presentation

### Case-1 [Generalised tonic-clonic seizures]

A 4-year-6-month-old black South African male with perinatal HIV infection presented with generalised tonic-clonic seizures, 3 months after initiation of EFV-based antiretroviral treatment (ART).

The child’s initial ARV regimen, started at 5 months of age, consisted of lamivudine (3TC), stavudine (d4T), and lopinavir/ritonavir (LPV/r) twice daily (bd). At 4 years of age he was enrolled in a treatment strategy trial evaluating ART switches in virologically suppressed children undertaken at Rahima Moosa Mother and Child Hospital in Johannesburg, South Africa (ClinicalTrials.gov, NCT01146873) [1]. Abacavir (ABC) was substituted for d4T and LPV/r was switched to EFV 8 weeks thereafter. EFV dosing was prescribed according to standard recommended weight-based dosing as per South African guidelines [2]. Baseline assessment for neuropsychiatric symptoms and neurological examination revealed no abnormalities.

Three months post-EFV initiation, the child was hospitalised for seizures. The mother reported that the child had multiple seizure episodes over a 3-day period with pyrexia. No further information was provided. Lumbar puncture (LP) showed no abnormalities and the child was discharged the following day on an unknown oral antibiotic and paracetamol. After the episode, the mother described the child’s behavior as becoming progressively more aggressive; fighting with his siblings; defiant towards his parents, but never violent. Routine clinical examinations revealed no CNS abnormalities.

Ten months after switching to EFV, the child experienced a generalised tonic-clonic seizure while being seen at a routinely scheduled study visit. The seizure lasted for approximately 1 minute, with no urination, tongue-biting, hypersalivation or obvious postictal confusion. The child had been seizure-free and clinically well since his prior hospital admission. In addition to EFV-based ART, tetryzoline (Spersallerg®) eye drops had been prescribed for allergic conjunctivitis. No known allergies or intolerances were reported.

After the witnessed seizure the child was hospitalised, and investigations including a chest x-ray (CXR), urine dipstick, septic blood work-up and LP revealed no abnormalities. The child was discharged the following day with a diagnosis of idiopathic childhood epilepsy and started on an antiepileptic drug (AED), sodium valproate, 15 mg/kg/dose once daily (od). Outpatient electroencephalogram (EEG) revealed no abnormalities and a contrast Computed Tomography brain (CTB) scan showed normal developmental structures, with no lesions or mass effect.

Plasma EFV levels were subsequently measured and genotyping performed as part of clinical care in an attempt to understand the nature of the presenting problem. The plasma EFV level, 13 h post dose, was >20.0 mg/L (suggested reference range: 1–4 mg/L) [3] as a result of the genetic characterisation which showed the child to be heterozygous for both *CYP2B6 c.516G/T* and *c.785A/G* genotypes. Consequently, EFV was discontinued and LPV/r restarted. The AED was stopped after 7 weeks. No further seizures have been reported, 30 months post-EFV discontinuation, and the aggressive behavior has improved.

### Case-2 [Cerebellar dysfunction]

A 4-year-10-month-old black Zimbabwean female with perinatal HIV infection presented with clinical findings consistent with cerebellar dysfunction, 20 months after being switched to an EFV-based ART regimen.

She was initiated on 3TC, d4T and LPV/r bd at 10 weeks of age. Achieving and maintaining viral suppression, at 37 months of age she was enrolled in the same clinical trial described above [1] and randomised to substitute ABC for d4T and EFV for LPV/r 1 month thereafter. EFV dosing was prescribed according to standard recommended weight-based dosing as per South African guidelines [2]. Baseline assessment for neuropsychiatric symptoms and neurological examination revealed no abnormalities.

Nineteen months after switching to EFV-based ART the child presented with ataxia and tremors. The EFV dose had been increased from 200 mg to 300 mg od 1 week prior to presentation, as part of routine weight-based dose adjustment. At this event, antibiotics were prescribed for a urinary tract infection, and an outpatient CTB scheduled, but was never performed.

Six weeks after the initial presentation the child was noted as having progressively worsening ataxia, marked upper and lower limb tremors and head bobbing. She also complained of epigastric pain and post-prandial vomiting. Examination on hospital admission revealed an ataxic gait, titubation, dysarthria, intention tremor, increased tone with cog-wheel rigidity in all limbs, and truncal hypotonia. Power and reflexes in upper and lower limbs were assessed as normal.

An urgent CTB scan revealed no abnormalities and a follow-up Magnetic Resonance Imaging scan was within normal limits. Laboratory findings including urea, electrolytes, full blood count, C-reactive protein (CRP) all reported within normal limits, and blood culture results showed no bacterial growth. Cerebrospinal fluid analysis was within normal limits. Further investigations, including screening for inborn errors of metabolism, were unable to be carried out as the parents refused further hospital admission.

Follow-up 2 months post-admission showed persistence of the CNS signs and symptoms. At this time, EFV toxicity was considered as a possible cause of the cerebellar dysfunction, drug levels and genotyping were obtained and EFV was replaced with LPV/r.

Mid-dosing plasma EFV levels, taken 13 h post dose, were reported at 60.54 mg/L (suggested reference range: 1-4 mg/L) [3] resulting from *CYP2B6* genotyping, where the child was found to be heterozygous carrying both *CYP2B6 c.516G/T* and *c.983 T/C* genotypes.

Upon EFV discontinuation, significant improvement of the cerebellar signs and symptoms was noted. Follow up was for a period of 2 months, when the family relocated, 1 month of clinical observation with the caregivers reporting continued clinical improvement telephonically thereafter.

### Case-3 [Generalised tonic-clonic seizures]

A 7-year-6-month-old black South African female with perinatal HIV infection presented with generalised tonic-clonic seizures 14 months after starting an EFV-based regimen.

The child was enrolled in an earlier trial at the same site (ClinicalTrials.gov, NCT00117728) [4] and initiated on 3TC, d4T and LPV/r at 17 months of age. Having achieved viral suppression, she was randomised to substitute NVP for LPV/r 7 months after ART initiation. The diagnosis of pulmonary tuberculosis necessitated discontinuation of NVP and re-introduction of LPV/r at age 3 years 10 months. She was subsequently enrolled in a follow up trial [1] and at age 6 years 4 months, where LPV/r was randomised to EFV. EFV dosing was prescribed according to standard recommended weight-based dosing as per South African guidelines [2]. Baseline assessment for neuropsychiatric symptoms and neurological examination revealed no abnormalities.

Thirteen months after starting EFV-based ART, the child was hospitalised with generalised tonic-clonic seizures and a suspected diagnosis of meningoencephalitis and tonsillitis. Neurologically she had a diminished level of consciousness (Glasgow coma scale of 10/15), meningism, increased tone, and brisk reflexes globally. An urgent CTB scan revealed no abnormalities. Besides an elevated CRP, her blood and LP results were within normal limits. On reassessment the following day she was awake, alert and fully cooperative with no meningism, and normal tone and reflexes.

Anti-epileptic therapy, sodium valproate controlled release (CR) tablets 200 mg bd, were started and EFV was continued. She was also discharged on paracetamol and multivitamin. Sodium valproate CR was stopped when the prescription was inadvertently discontinued after 1 month.

The child was admitted 2 years later with her second seizure episode having taken EFV-based ART for a 3-year period. Although two further seizure episodes were reported since her prior admission, no medical attention had been sought. Seizures were described by the caregiver as generalised tonic-clonic in nature accompanied by urinary incontinence and a distinct postictal period. On admission the child was apyrexial with a normal physical examination except for enlarged uninfected tonsils. An elevated CRP and white blood cell count were noted, but other investigations were within normal limits. No LP was performed. She was restarted on anticonvulsants, but defaulted treatment after 2 months. An outpatient CTB was found to be normal.

Plasma EFV concentration was measured as a suspected cause of the seizure activity. Plasma EFV level, 14 h post-dose, was 51.23 mg/L (suggested reference range 1-4 mg/L) [3] as a result of *CYP2B6* genotyping revealing heterozygosity for *CYP2B6 c.516G/T* and *c.983 T/C* genotypes. EFV was discontinued and replaced with LPV/r. Follow-up, 14 months post EFV-discontinuation, has revealed no abnormalities and no further seizures were reported.

### Case-4 [Absence seizures]

As previously described by Strehlau et al., a 4-year-7-month-old black South African female presented with new onset absence seizures and behavioral changes 1 month after starting an EFV-based regimen [5].

## Conclusions

For more than a decade, EFV, in combination with a non-nucleoside reverse transcriptase inhibitor backbone, has been recommended as part of the first-line ART regimen for adults and children older than 3 years in South Africa [2, 6–8]. EFV is an attractive drug for the management of children infected with HIV owing to once-daily dosing, high potency, child-friendly formulations, palatability, and alignment with adult regimens. When compared to NVP in a meta-analysis of adults and children on first-line therapy, EFV was observed to result in fewer treatment discontinuations despite some patients presenting with severe CNS effects [9]. In addition to cutaneous side effects, EFV is most commonly associated with early, mild, transient nervous system side effects in both adults [3, 9–15] as well as children [9, 12, 16]. Dizziness, headaches, nightmares and difficulty sleeping, tend to resolve spontaneously within the first month of treatment. Severe CNS adverse events appear to be infrequent and are not well described in children.

A number of cases of severe adverse CNS side effects associated with EFV use in children have been reported. A 10-year-old girl experienced one episode of generalised seizures 6 weeks after switching from a PI-based to an EFV-based regimen. It was noted that the patient had a strong family history of epilepsy, although she had not previously experienced seizures. ART was not interrupted, she was not initiated on anticonvulsants and the seizures did not recur [17]. Another reported case of a 12-year-old girl presenting with psychosis associated with long term EFV use with genetic analysis showing a heterozygous *CYP2B6 c.516G/T* genotype [18]. Two of the 33 children (6 %) reported on in an EFV pharmacokinetics study, presented with adverse events, namely a psychotic reaction and seizures, with no further details provided [19].

In this case series, we describe severe CNS adverse events in four children between 4 and 8 years of age presenting between 1 and 20 months post-EFV initiation. In all four cases the plasma EFV levels were substantially higher than the upper limit of the therapeutic range (1–4 mg/L) [3]. Children in this series presented with a variety of abnormal CNS signs and symptoms – absence seizures, generalised seizures, and cerebellar dysfunction. We believe this is the first case of cerebellar dysfunction associated with EFV treatment to be described in a child. Furthermore, in three of the cases, CNS sequelae manifested late, 3–20 months, after switching to EFV. All four cases showed significant improvement, and even resolution of the CNS abnormalities, once EFV treatment was discontinued.

EFV is primarily metabolised by the CYP2B6 enzyme and to a lesser extent by CYP3A5, CYP3A4, CYP2A6 and CYP1A2 isoforms of the cytochrome P450 system in the liver [20, 21]. The gene coding for CYP2B6 is highly polymorphic and the *CYP2B6 c.516G > T* SNPs [11, 21–32] and *CYP2B6 c.983 T > C* SNPs [23, 25, 33–37] have been reportedly associated with reduced EFV oral clearance resulting in increased plasma EFV concentrations. The single nucleotide variant *CYP2B6* *c.516 T* is linked to a CYP2B6 mRNA splice variant that lacks exons four to six and consequently lower levels of functional CYP2B6 enzyme [24], while the *CYP2B6 c.983C* variant causes a non-synonymous amino acid change from isoleucine to threonine at position 328 in exon 7 and, thus, reduced CYP2B6 activity [37]. Furthermore it is known that significant differences in allele frequencies between different populations exist, for example the frequency of the *CYP2B6 c.516 T* allele ranges from 14 % in Koreans [38] to 27–30 % in Caucasians [36], 49 % in Africans [39] and 62 % in Papua New Guineans [40]. The *CYP2B6 c.983C* allele is, however, not observed in Asian or Caucasian individuals, but is present in 4–9 % of Africans and African-Americans [41].

The child in case 4 was homozygous for *CYP2B6 c.516 T/T* which has been shown to be associated with substantially decreased CYP2B6 mRNA expression and therefore higher plasma EFV levels leading to an increased risk of CNS toxicity [3]. In a study done by Swart et al. [36], the frequencies of *CYP2B6 c.516G/T* and *T/T* genotypes among healthy, black South Africans were 0.48 and 0.13, respectively. In the same study 88 % of patients with the *CYP2B6 c.516 T/T* genotype presented with plasma EFV concentrations above the therapeutic range. A significant number of South African HIV-infected patients could potentially benefit from EFV dosage optimisation or reduction.

Both children in cases 2 and 3 carried *CYP2B6 c.516G/T* and *c.983 T/C* genotypes, and individuals with dual *c.516G/T – c.983 T/C* heterozygosity are CYP2B6 slow metabolisers, because *CYP2B6 c.516 T* and *CYP2B6 c.983C* variants reside on mutually exclusive haplotypes. [42] Carriers of both the *CYP2B6 c.516 T* and *c.983C* variants are more likely to present with high plasma EFV levels and exhibit poorer CNS responses [23, 25, 33, 37]. *CYP2B6 c.983 T/C* and *C/C* genotypes are present in 5–11 % and 0–2 %, respectively, of black South Africans [36]. Swart et al. [36], reported on nine adult patients with both the *c.516G/T* and *c.983 T/C* genotypes with an average plasma EFV concentrations >12 mg/L.

The child described in case 1 was heterozygous for *CYP2B6 c.516G > T* and the *CYP2B6 c.785A > G* SNP. Similar to other populations, *CYP2B6 c.516G > T* SNP is in tight linkage disequilibrium with the *c.785A > G* SNP. Genotyping for the *c.785A > G* SNP, which is located in exon 5 of CYP2B6, is thus not necessary [43, 44]. Studies have reported a gene-dose effect with EFV clearance following the pattern T/T < G/T < G/G and EFV levels following the pattern T/T > G/T > G/G [27, 29]. This could explain the lower EFV level in this case, however, genotyping for *CYP2B6* c.*983 T/C* was not performed.

EFV use has been well studied in adult populations, but less so among children where physiological changes may further complicate drug metabolism. For example, hepatic enzyme activity is increased between the ages of 1–4 years, potentially impacting drug metabolism [45]. Findings from Saitoh et al. [27], suggest that age may need to be considered when evaluating the impact of genetic variants on antiretroviral pharmacokinetics in children.

With the increasing use of ART resulting in HIV becoming a chronic disease, and the extended duration perinatally infected children will remain on treatment, reducing long-term ART-related side effects is a priority. A reduced EFV dose of 400 mg from 600 mg in adults was found to be non-inferior with a modest improvement in adverse events [46]. Similarly, an EFV pharmacokinetics study in Ugandan adults suggested that a daily EFV dose of 300 mg may be adequate for individuals carrying the *CYP2B6 c.516 T/T* genotype [47]. Ter Heine et al. [28], showed that children carrying the *CYP2B6 c516G/G* genotype had a 50–70 % probability of developing sub-therapeutic EFV concentrations, pointing towards the need for dose optimisation in both adults and children. Cases 1, 2 and 3, as compared to case 4, carried not only the *CYP2B6 c. 516G > T* SNP but additional heterozygous SNP’s conferring impaired *CYP2B6* activity resulting in higher plasma EFV concentrations. Thus, screening for potential EFV-toxicity based on the *CYP2B6 516* SNP alone would fail to predict the patients with severely impaired EFV metabolism. We hypothesize that genotype-assisted EFV dose optimisation could possibly assist with a reduction in severe CNS toxicities. Both *CYP2B6 c.516G > T* and *c.983 T > C* SNP’s should be considered in predicting EFV plasma levels pronounced effects, in decreasing the enzyme activity and frequencies of the allelic variants among African populations. While further research characterising the full spectrum of adverse events associated with EFV are needed, clinical vigilance and a high level of suspicion with any reported neurobehavioral or CNS abnormalities for children receiving EFV-based regimens are needed.

## Consent

Genetic characterization was performed as part of the clinical evaluation in each case. Verbal informed consent was obtained from patients’ caregivers before testing. Written informed consent was obtained from all patients’ caregivers to collate and publish information found in this case report. A copy of the written consent is available for review by the editor of this journal.

## Abbreviations

3TC: lamivudine
A: adenine
ABC: abacavir
AED: anti-epileptic drug
ART: antiretroviral therapy
ARV: antiretroviral
bd: twice-daily dosing
C: cytosine
CNS: central nervous system
CR: controlled release
CRP: C-reactive protein
CTB: computed tomography brain
CXR: chest x-ray
CYP2B6: cytochrome P450 2B6
d4T: stavudine
EEG: electroencephalogram
EFV: efavirenz
G: guanine
HIV: human immunodeficiency virus
LP: lumbar puncture
LPV/r: lopinavir/ritonavir
NNRTI: non-nucleoside reverse transcriptase inhibitor
od: once-daily dosing
SNP: single nucleotide polymorphism
T: thymine
